# TIM-3 expression in human osteosarcoma: Correlation with the expression of epithelial-mesenchymal transition-specific biomarkers

**DOI:** 10.3892/ol.2013.1410

**Published:** 2013-06-18

**Authors:** YONGJUN SHANG, ZHANYONG LI, HONG LI, HAIBO XIA, ZHENHUA LIN

**Affiliations:** 1 Department of Pathology and Cancer Research Center, Yanbian University Medical College, Yanji, Jilin 133002;; 2 Department of Orthopedics, Affiliated Hospital of Chifeng University, Chifeng, Inner Mongolia 024000;; 3 Department of Otorhinolaryngology and Head-Neck Surgery, Xinqiao Hospital, PLA Third Military Medical University, Chongqing 400037, P.R. China

**Keywords:** osteosarcoma, T cell Ig- and mucin-domain-containing molecules, immunohistochemistry, tumor pathogenesis

## Abstract

Signals from the T cell Ig- and mucin-domain-containing molecules (TIMs) have been demonstrated to be actively involved in regulating the progression of carcinomas. However, the expression and distribution of these molecules in osteosarcoma, the most common primary bone malignancy with poor prognosis, have not been investigated. In this study, the expression of TIMs was examined in nine invasive human osteosarcomas using immunohistochemistry, and the phenotypes were detected by dual immunofluorescence staining. Using immunohistochemistry, it was observed that only TIM-3, rather than TIM-1 or TIM-4, was expressed in these tumor specimens, where it was localized in the cytoplasm and plasma membrane of tumor cells. Dual immunofluorescence staining revealed that the expression of TIM-3 was observed in all cell types investigated, including CD68^+^ macrophages, CD31^+^ endothelial cells, CK-18^+^ epithelial cells and PCNA^+^ tumor cells. Notably, in sarcoma cells, TIM-3 was co-expressed with certain biomarkers of epithelial-mesenchymal transition (EMT), including vimentin, Slug, Snail and Smad. These combined results suggest that TIM-3 triggers tumor cells to acquire features of aggressive EMT and may be involved in the pathogenesis of this malignancy.

## Introduction

Osteosarcoma is the most common type of non-hematopoietic primary malignant bone tumor. Following the initial diagnosis, patients are usually treated with multi-agent preoperative chemotherapy and surgical resection, followed by postoperative chemotherapy. Despite significant progress in chemotherapy, patients who have metastases at diagnosis continue to have poor prognoses (1). Therefore, it is essential to identify additional biomarkers for use in diagnosis and novel therapeutic strategies.

T cell Ig- and mucin-domain-containing molecules (TIMs) are a recently described family. Three members of which, TIM-1, TIM-3 and TIM-4, have been identified in humans (2). TIM-1 was demonstrated to be preferentially expressed on Th2 cells and to support T-cell activation, promoting the pathogenesis of asthma and allergies (3–5). TIM-4 was observed on antigen-presenting cells (APCs) and mediates the phagocytosis of apoptotic cells (6). By contrast, TIM-3 was originally identified as a surface molecule expressed on CD4^+^ Th1 cells. The interaction of TIM-3 with its potential ligand, galectin-9, induces Th1 cells to undergo apoptosis and inhibits their production of IFN-γ (7–9).

In addition to activated T cells, ectopic expression of TIMs has been observed on tumor cells, where they were also described as being actively involved in the pathogenesis of tumor development. For example, non-small cell lung cancer patients whose tumor tissues were positive for TIM-3 had significantly shorter survival times compared with those with TIM-3- tumor tissues (10). In patients with hepatitis B virus-associated hepatocellular carcinoma, the number of TIM-3^+^ infiltrating tumor cells was negatively associated with patient survival (11). Moreover, TIMs were also shown to function as specific markers for the diagnosis of Langerhans cell sarcoma, head and neck cancer and follicular B cell non-Hodgkin lymphoma (12,13), while the combined blockade of TIM-3 and TIM-4 augments the efficacy of cancer vaccines against established melanomas (14).

Epithelial-mesenchymal transition (EMT) is an essential process for normal development and is also crucial in cancer dissemination, endowing cells with metastatic and cancer stem cell properties (15). EMT is characterized by the down-regulation of epithelial markers (such as E-cadherin) and the upregulation of vimentin, as well as other mesenchymal markers, resulting in numerous phenotypic changes, including the loss of cell-cell adhesion and cell polarity and the acquisition of migratory and invasive properties (16). TWIST1, SNAIL and SLUG are transcription factors that govern EMT and are regulated by TGF-β and microRNA (17,18). The increased expression of EMT biomarkers has been associated with poor prognostic clinicopathological features of various types of cancer, including osteosarcoma (19–21).

In the present study, the expression of TIMs in osteosarcoma samples was analyzed by immunohistochemistry, and the associations between TIMs and EMT biomarkers were further analyzed by dual immunofluorescence staining.

## Materials and methods

### Patients

**Figure 1.**
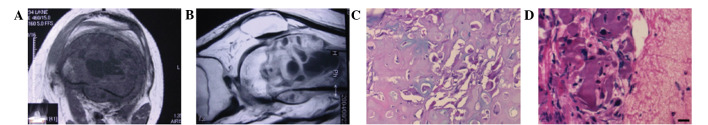
Histopathology and pathological characteristics of osteosarcoma were analyzed by CT examination and H&E staining. (A) Axial and (B) sagittal CT images demonstrating that tumor development arose from the bone. (C) Histopathology and (D) pathological characteristics of osteosarcoma from two patients were detected by H&E staining. Scale bar, 20 *μ*m.

Samples from nine cases of osteosarcoma were collected at the Department of Orthopedics, Affiliated Hospital of Chifeng University (Chifeng, China). The histopathology and pathological characteristics of the patients were analyzed by CT examination and H&E staining, with the results demonstrating that all patients had osteosarcoma (Fig. 1). All osteosarcoma samples were obtained from the legs, and the tissues were fixed in 10% neutral buffered formalin, and then embedded in paraffin. This study protocol was approved by the review board of the ethics committee of Chifeng University. Written informed consent was obtained from all patients.

### Immunohistochemistry

The immunohistochemistry was performed as published previously, but with slight modifications (22). Briefly, paraffin-embedded tissue blocks were cut into 2–3-*μ*m sections and mounted on poly-L-lysine-charged glass slides (Sigma, St. Louis, MO, USA). After the sections were dewaxed and rehydrated, antigen retrieval was performed by microwaving in 10 mM citrate buffer (pH 6.0). The sections were cooled to room temperature (RT) and endogenous peroxidase activity was blocked by incubation with a solution of 0.5% hydrogen peroxide in 50% methanol for 1 h. The sections were then incubated in 3% BSA (Sigma) with 0.1% Nonidet P-40 (Beyotime, Haimen, China) in PBS (Wuhan Boster Biological Technology Ltd., Wuhan, China) for 1 h at RT to block nonspecific binding. Subsequently, the sections were incubated overnight at 4°C with primary antibodies (Table I), including anti-TIM-1, anti-TIM-3 or anti-TIM-4, diluted in 1% BSA. After washing, the sections were incubated with the corresponding secondary antibodies for 1 h at RT. The Vectastain ABC kit (Vector Laboratories, San Diego, CA, USA) was used for the avidin-biotin complex method according the manufacturer’s instructions. Sections incubated with isotype-matched, concentration-matched Ig without primary antibodies were used as isotype controls. Peroxidase activity was visualized with the DAB Elite kit (K3465; Dako, Copenhagen, Denmark) and brown coloration of tissues represented positive staining. The sections were lightly counterstained with hematoxylin, dehydrated through an ethanol series, cleared in xylene and mounted. Subsequently, the sample sections were viewed using a light microscope (Axioplan 2; Zeiss, Berlin, Germany).

### Dual immunofluorescence staining

For dual immunofluorescence staining, the sections were incubated with primary anti-TIM-3 antibodies at 4°C overnight. After washing with PBS (three washes, 5 min per wash), the sections were incubated with Alexa Fluor^®^ 555-conjugated goat anti-mouse/rabbit IgG antibodies (Invitrogen, Carlsbad, CA, USA) for 1 h. The sections were further incubated with anti-CD68, anti-CD31, anti-PCNA, anti-Bcl-2, anti-Snail, anti-Slug, anti-Smad, anti-pSmad2/3 or anti-CK-18 antibodies at 4°C overnight, and incubated with Alexa Fluor^®^ 488-conjugated goat anti-mouse/rabbit IgG1 antibodies (Invitrogen) for an additional hour. Subsequently, the sections were incubated with 1 *μ*g/ml DAPI (Sigma) for 10 min to stain the nuclei. Sections incubated with the appropriate isotype control primary antibodies and fluorescently labeled secondary antibodies were used as negative controls. The results were analyzed by fluorescence microscopy (Axioplan 2; Zeiss).

## Results

### Expression and anatomical distribution of TIMs in sections from osteosarcoma

**Figure 2.**
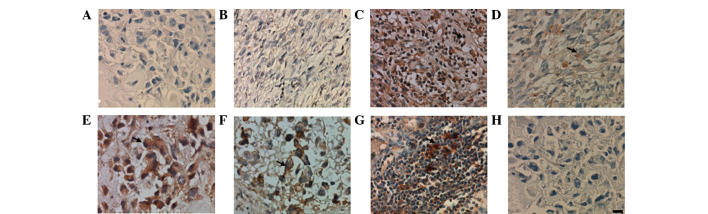
Expression of TIM-1, TIM-3 and TIM-4 in osteosarcoma sections as detected by immunohistochemistry. (A) Goat IgG isotype control antibodies showed no positive staining; (B) TIM-1 was absent in sections from osteosarcoma patients. TIM-3 was expressed on (C) infiltrating lymphocytes, (D) macrophages and (E and F) tumor cells in sections from osteosarcoma patients. TIM-4 was expressed on (G) macrophage-like cells while it was absent from (H) tumor cells. Arrows indicate the positive cells. Scale bar, 20 *μ*m. TIM, T cell Ig- and mucin-domain-containing molecule.

Axial and sagittal CT images demonstrated that tumor development originated from the bone (Fig. 1A and B). The specimens from all nine cases of osteosarcoma were highly cellular tumors consisting of enlarged round cells. The neoplastic cells showed the presence of cytological atypia, with multiple hyperchromatic and prominent nucleoli (Fig. 1C and D). Immunohistochemistry showed that TIM-3^+^ and TIM-4^+^, but not TIM-1^+^, cells were observed in all cases. These molecules were located on cell membranes and in the cytoplasm. However, the distributions of TIM-3 and TIM-4 were noticeably different. TIM-3 was identified on macrophages (Fig. 2A), infiltrated inflammatory cells (Fig. 2B) and tumor cells (Fig. 2C and D), and TIM-3^+^ cells were distributed throughout the tissue sections. TIM-4, however, was expressed on macrophage-like cells (Fig. 2E) and was absent from tumor cells (Fig. 2F). No positive results were observed in sections incubated with only secondary antibodies (goat IgG1), which were used as the negative controls (data not shown).

### Phenotypes of TIM-3 in sections from osteosarcoma

**Figure 3.**
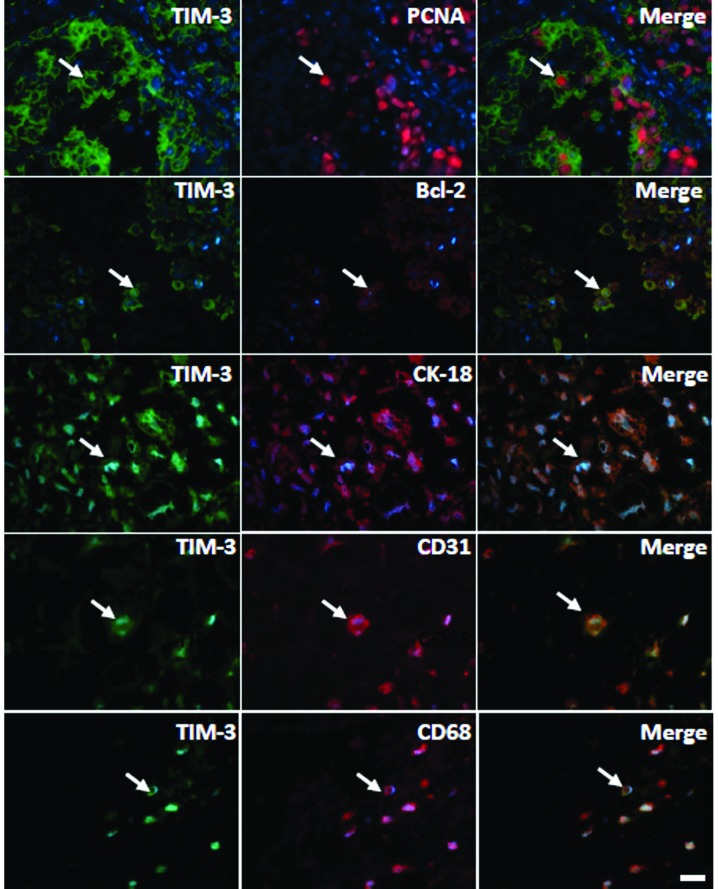
Morphology of TIM-3^+^ cells in osteosarcoma sample sections as detected by dual immunofluorescence staining. Dual immunofluorescence staining showed that TIM-3 was expressed on CD68^+^ macrophages, CD31^+^ endothelial cells, CK-18^+^ epithelial cells, Bcl-2^+^ tumor cells and PCNA^+^ tumor cells. Arrows indicate positive cells. Nuclei were stained with DAPI. Scale bar, 20 *μ*m. TIM, T cell Ig- and mucin-domain-containing molecule.

As TIM-3 was observed in tumor cells from osteosarcomas, the phenotypes of the TIM-3^+^ cells were further examined by dual immunofluorescence staining. As shown in Fig. 3, TIM-3 was expressed on CD31^+^ endothelial cells, CK-18^+^ epithelial cells and CD68^+^ macrophages. In addition, TIM-3 was co-expressed with Bcl-2 and PCNA, indicating that TIM-3 may regulate tumor cell apoptosis and proliferation (Fig. 3).

### Associations between TIM-3 and EMT biomarkers in sections from osteosarcoma

**Figure 4.**
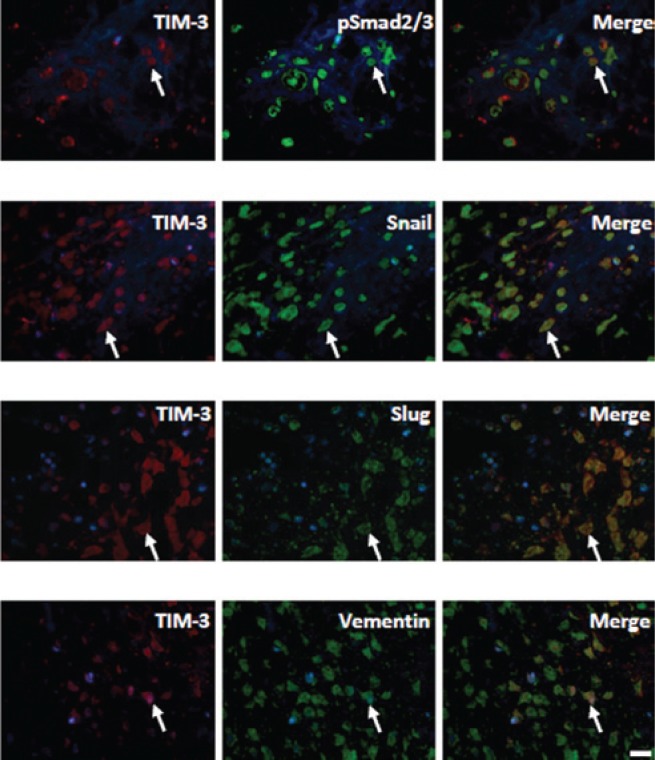
Association between the expression of TIM-3 and certain bio-markers of EMT in osteosarcoma sections detected by immunofluorescent dual staining. Dual immunofluorescence staining showed that the expression of certain biomarkers of EMT, including Slug, Snail and Smad, were co-expressed with TIM-3 in the same carcinoma cells. Nuclei were stained with DAPI. Scale bar, 20 *μ*m. EMT, epithelial-mesenchymal transition. TIM, T cell Ig- and mucin-domain-containing molecule.

Previous studies demonstrated that the expression of EMT biomarkers, including Slug, Snail and Smad, could be detected in samples from patients with osteosarcoma, suggesting that EMT may be involved in the pathogenesis of osteosarcoma (23,24). In the present study, the correlations between TIM-3 and these EMT biomarkers were also investigated. The results showed that TIM-3 was co-expressed with Slug, Snail and Smad in the same sarcoma cells (Fig. 4).

## Discussion

Osteosarcoma, derived from primitive mesenchymal cells and originating from bone, is the most common type of primary bone tumor in children and adolescents. Characteristically, this sarcoma occurs frequently in the metaphyseal regions of long bones and metastasizes preferentially to the lung (1,25). Although it is a relatively uncommon type of cancer, the incidence is increasing (26,27). With advances in multimodal treatments consisting of adjuvant chemotherapy and surgical resection, the prognosis and quality of life of patients with non-metastatic osteosarcoma of the extremities are greatly improved. Nevertheless, the five-year progression-free survival of high-grade osteosarcoma is only ∼50% due to the failure of rescue chemotherapy (28). Therefore, it is necessary to establish new therapeutic strategies to improve the overall rate of survival.

TIMs are proteins that are actively involved in tumor development, in addition to the pathogenesis of rheumatoid arthritis, asthma, systemic lupus erythematosus, multiple sclerosis and diabetes (2). TIMs have been reported to be aberrantly expressed in carcinoma tissues, and the presence of TIM-3 modulates tumor development and carcinoma traits (3). In the present study, the expression of TIMs was analyzed in nine cases of osteosarcoma and the results demonstrated that only TIM-3, rather than TIM-1 or TIM-4, was detected on the tumor cells of osteosarcoma patients. Notably, the morphological analysis demonstrated that TIM-3 was also present on CD68^+^ macrophages, CD31^+^ endothelial cells, CK-18^+^ epithelial cells, as well as on Bcl-2^+^ and PCNA^+^ tumor cells. These results suggested that TIM-3 is involved in the progression of osteosarcoma via the promotion of tumor cell proliferation, as well as the inhibition of apoptosis.

In patients with advanced cancer, widespread manifestation of distant metastases is a major cause of cancer-associated mortality. Despite this important clinical problem, little is known about the mediators that promote tumor outgrowth in the metastatic organ. At present, the transdifferentiation of polarized epithelial cells to mesenchymal cells (EMT), which occurs during tumor invasion and metastasis, is recognized as a key developmental process (29). The acquisition of invasiveness by cancer cells through the invasion and destruction of the basement membrane is considered to represent the onset of a multistep process that eventually leads to metastatic dissemination with life-threatening consequences. In addition to promoting tumor cell invasion and metastasis, EMT generates cancer cells with stem cell-like characteristics, including increased self-renewal and tumor-initiating capabilities, as well as increased resistance to apoptosis and chemotherapy (30). Previous studies have examined the expression of EMT biomarkers, such as Slug, Snail and Smad, in osteosarcoma sections, suggesting that EMT may be involved in the pathogenesis of osteosarcoma (23,24). The present study also detected correlations between TIM-3 expression and the expression of these EMT biomarkers. The results showed that TIM-3 was co-expressed with Slug, Snail and Smad in the same sarcoma cells, suggesting that TIM-3 may trigger the process of EMT to promote tumor development. However, the exact mechanism of this requires further investigation.

In summary, the present study investigated the expression of TIMs in sections from osteosarcoma patients and an understanding of the functional roles of TIM-3 may aid in the development of novel strategies for disease diagnosis or immunotherapy.

## Figures and Tables

**Table I.** Immunohistochemical study: Antibodies, source and dilution.

Ab	Dilution	Clone	Source
TIM-1	1:100	Polyclonal Goat IgG	R&D System
TIM-3	1:100	Polyclonal Goat IgG	R&D System
TIM-4	1:100	Polyclonal Goat IgG	R&D System
CD3	1:50	Monoclonal mouse IgG (F7.2.38)	Dako
CK-18	1:400	Monoclonal mouse IgG (C-04)	Santa Cruz
CD68	1:50	Monoclonal mouse IgG (3F103)	Santa Cruz
CD31	1:50	Monoclonal mouse IgG (10G9)	Santa Cruz
CD1a	1:200	Monoclonal mouse IgG (7A7)	Abcam
PCNA	1:200	Monoclonal mouse IgG (F-2)	Santa Cruz
Bcl-2	1:100	Monoclonal mouse IgG (C-2)	Santa Cruz
Slug	1:100	Monoclonal mouse IgG	Abcam
Snail	1:100	Polyclonal Goat IgG	Abcam
Smad2	1:100	Polyclonal Goat IgG	Santa Cruz
Smad3	1:100	Polyclonal Rabbit IgG	Santa Cruz
Vimentin	1:50	Monoclonal mouse IgG (RV202)	Santa Cruz
E-cad	1:200	Polyclonal Rabbit IgG	Santa Cruz
